# Social networks of neighbourhood inhabitants, residents of a care facility, and nursing staff: a case study in two long-term care facilities in the Netherlands

**DOI:** 10.1186/s12877-025-05948-z

**Published:** 2025-05-08

**Authors:** Adriana P. A. van Beek, Suzanne Portegijs, Peter P. Groenewegen, Martine W. J. Huygens, Beate G. M. Völker

**Affiliations:** 1 Netherlands Institute for Healthcare Services Research (Nivel), Utrecht, The Netherlands; 2 Viva! Zorggroep, Care Organization, Velsen, The Netherlands; 3 Department of Human Geography and Spatial Planning and Department of Sociology, Utrecht University, Utrecht, The Netherlands; 4 Netherlands Institute for the Study of Crime and Law Enforcement (NSCR), Amsterdam, The Netherlands; 5 Department of Human Geography and Spatial Planning, Utrecht University, Utrecht, The Netherlands

**Keywords:** Long-term care, Social networks, Community resources, Dementia

## Abstract

**Background:**

The pressure on long-term care (LTC) facilities, because of population ageing and personnel shortages, may be relieved by using the social network resources that are already available to the facility. The aim of this study is to give insight in existing social networks and relationships between residents and their family members, care staff and persons in the local communities of nursing homes.

**Methods:**

In this paper we describe these social networks and the relationships of which they consist in two nursing homes in the Netherlands, thereby illustrating the social capital of both facilities.

**Results:**

The results show there are multiple direct and indirect relationships between nursing staff, family members of residents and inhabitants of the neighbourhood.

**Conclusions:**

Although it may be difficult for residents with dementia to maintain their social networks as part of their illness, there are numerous ties that attest to the social roles of persons with dementia in the community. These ties can be used to provide person-centred care, but are also an important resource in finding and retaining personnel and volunteers.

**Supplementary Information:**

The online version contains supplementary material available at 10.1186/s12877-025-05948-z.

## Background

In the Netherlands – as in many other countries – the population is ageing and overall the need for care increases. This puts much pressure on long-term care (LTC) facilities such as nursing homes. We argue that this pressure can be reduced by using the social resources embedded in the networks of nursing homes. Nursing homes do not exist in a social vacuum but are connected to the local community [14]. Residents of nursing homes often used to live in the municipality where the facility is located, before admission [33]. Staff members, who work in nursing homes often have their home and family close to the facility, have direct and indirect informal relationships with neighbourhood inhabitants, or have professional contacts with nursing home residents and their informal carers. Importantly, residents of nursing homes and staff members may also be connected via common network members in the neighbourhood, directly or more indirectly, by knowing each other’s friends or family. These contacts are especially important in dementia care. Persons with dementia are, due to their illness, often unable to maintain their social ties. As a result, it is crucial for these patients’ wellbeing to maintain social roles and social support from others in the beginning of dementia [19], but also when they are admitted to a nursing home [12]. Such direct and indirect social ties between nursing staff and residents with dementia, increase the information nursing staff have about the residents and are thus important for person centred care [1].

Another important consequence of the network embeddedness of a facility is that quality of care may be enhanced through employment of social resources in relationships. Firstly, nursing homes need involvement of relatives and volunteers from the local community. Research showed that in 2019 about 80 percent of the residents of nursing homes in the Netherlands receive help from relatives and 43 percent from volunteers [33]. Relatives often help with domestic and personal care, while volunteers often offer company or emotional support, assist in indoor or outdoor activities, or assist in transport [17].

Secondly, facilities may be better able to attract and retain staff if they are integrated in the local community. Many facilities, such as hospitals, currently face difficulties in attracting enough and adequately skilled personnel [11, 31]. In the Netherlands, most employees in nursing homes are educated at intermediate vocational level. They often search for employment in the area where they are educated and live [32]. Employees who live near the workplace with many family ties or community commitments tend to stay longer with the organisation [24, 37].

More general, many studies have shown that the embeddedness of organisations crucially impacts their functioning. Since the work of Uzzi [30], multiple studies demonstrated the importance of embeddedness of organisations for organisational performance [5], while others showed that embeddedness also impacts organisational citizenship (e.g., Lee et al. 2004 [8]), absenteeism [21], and voluntary turnover [9, 34]. While there is much research on networks in and around organisations, research on social networks in and around institutions such as nursing homes is rare. Nursing homes differ from other organisations because of the frailty of the patients and an educationally diverse staff [29]. Care in Dutch nursing homes can be characterized as continuing, long-lasting, systematic and multidisciplinary care [28]. Nursing homes in the Netherlands provide care to people needing 24/7 assistance and care in sheltered housing. Caring for people with dementia in nursing homes is preferably provided in smaller scale living environments [35]. Specifically trained elderly care physicians provide medical care for nursing home residents,regular care activities are provided by care and treatment professionals, including registered nurses, certified nurse assistants and nurse aids [2, 35].

The aim of this paper is to describe the social networks between the local communities of two nursing homes and residents and nursing staff of these nursing homes. The focus of our study is on the dementia ward in each of the two facilities.

Our overall research question is:

*What are the network connections between staff members and residents with dementia of nursing homes with inhabitants of the local neighbourhood at the place where the facility is located?*

We will start with describing the networks connecting nursing staff and residents with dementia, either with residents with dementia directly or indirectly through family members or other persons in the community. These types of ties are important because it has been shown that they improve quality of care. In units where nursing staff reported ties with relatives and acquaintances of residents, nursing staff treated residents more often with respect and approached residents in a friendly manner both by being at ease and by starting friendly conservations with residents [1]. We will explore these connections in three steps. First by describing the connections between the facility’s staff members with the neighbourhood inhabitants. Next, we explore the connections of the facility residents with dementia with the neighbourhood. Finally, we will look at the opportunities that nursing homes provide to neighbourhood inhabitants to meet residents and staff through the provision of services at the premises.

The answer to our general question constitutes the basis for a further development of hypotheses about the resources in social networks of nursing homes and, more generally, LTC facilities, about the effects of the social embeddedness of LTC facilities, as well as about the way social embeddedness may be stimulated.

## Methods

### Aim

The aim of this study is to give insight in existing social networks and relationships between residents and their family members, care staff and persons in the local communities of nursing homes.

### Data collection

Data were collected in May and September 2018 in two nursing homes that provide dementia care in two small, rural villages in the south of the Netherlands.

Both villages have approximately 17.000 inhabitants and are each part of a larger municipality, consisting of several villages. One municipality is around 66 square kilometres; the other municipality is around 53 square kilometres. The municipalities respectively have around 24.000 and 28.000 inhabitants with a similar population density (more specifically 435 to and 450 inhabitants per square kilometre).

Data collection made use of standardized survey questions and entailed gathering information from neighbourhood inhabitants, nursing staff, and family members of residents with dementia. The questionnaires were developed for this study in collaboration with the facilities.

#### Questionnaire for nursing staff

The questionnaire for nursing staff was based on earlier studies on social networks of nursing staff in nursing homes, *see Appendix 1* [1]. Nursing staff members were asked if they were born in the village and if they currently lived in the village. In addition, we asked whether residents of the neighbourhood talked to them about the nursing home and about their conversations with family members of patients with dementia. In addition, we asked about existing ties between nursing staff and patients with dementia through relatives and acquaintances of patients with dementia in the community.

#### Questionnaire for the neighbourhood inhabitants

This questionnaire was developed in cooperation with the two nursing homes, *see Appendix 2*. It asked how often neighbourhood inhabitants visited the nursing home, the aim of their visits, if they knew persons living in the home, in what relation and how often they visited these persons. Regarding staff, we asked if neighbourhood residents knew staff members, the type of relationship and if they worked as a volunteer in the facility. In addition, to gain insight in the connectedness to the neighbourhood we asked if they were born in the village were the nursing home was located and how they viewed the neighbourhood they lived in. Demographic questions focused on gender, age, and education-level. In total, 500 questionnaires were distributed together with an information letter in the neighbourhoods of the two nursing homes: 250 for each facility. We identified the 500 neighbourhood inhabitants by making a printout of Google maps of the nursing home at the time of data collection and identifying 250 houses in the streets in a circle around each nursing home. Those houses received a questionnaire; the researchers walked the neighbourhood to distribute the surveys in the mailboxes. One questionnaire for each house. When all 250 questionnaires were distributed, we stopped. Questionnaires were distributed at one time only. A paper based questionnaire with a return-envelope was deemed most feasible as we did not have names or email addresses of neighbourhood.

#### Questionnaire for family members of residents with dementia

We asked family members how often they talked to nursing staff when they visited the nursing home and what they talked about, *see Appendix3*. We also asked if they talked to staff members on other occasions and if they knew staff before the patient with dementia was admitted to the nursing home. In addition, we asked if family members were born in the village, and if they lived in the village at this moment.

### Analysis

In this article we describe the networks between residents, staff, and inhabitants of the neighbourhood. We use the answers to open questions to characterise the direct and indirect ties between nursing home staff on the one hand and residents and their relatives on the other hand. Data were analysed using descriptive statistics in Stata.

### Research ethics

All persons participated in the study on a voluntary basis and after they were informed about the study. The questionnaires for community members were delivered by the researchers in the mailbox of their homes. As part of other studies within this project, information letters and informed consent forms for participation within this study were send by mail to the first legal representatives of all residents of the included wards by the care organizations. If the first legal representative agreed on participation the informed consent forms were signed and send back to the researchers. Only first legal representatives whom gave informed consent received a questionnaire for this particular study. Respondents could send the completed questionnaire to the research team by freepost. There was no obligation to fill out the questionnaires and respondents could stop at any moment without any consequences. There were no exclusion criteria for participation. Data was analysed anonymously. The study was undertaken in accordance with the declaration of Helsinki, the Code of Conduct for Health Research (code-of-conduct-for-health-research.eu). The overall research project to which this study belongs has been approved by the Medical Ethical Committee of the University Medical Centre Utrecht, the Netherlands (MEC number: 18–127/C).

## Results

### Participation in the study

The questionnaire for nursing staff was completed by 38 staff members of the two dementia wards in the two nursing homes. In total, 141 and 119 questionnaires for neighbourhood inhabitants were returned resulting in a response rate of 56% (nursing home) and 48% (nursing home 2).

The questionnaire for family members was returned by 9 respondents in facility 1 (32%), and 16 respondents in facility 2 (46%).

Descriptive demographics of the responders to the questionnaire among nursing home staff (*n* = 38) can be found in Table 1. Nursing staff in both nursing homes were around 46 years old and respondents were mostly female (respectively 94% and 95%). On average, respondents were working more than eight years in both nursing homes The descriptive demographics of the respondents to the neighbourhood resident’s questionnaire (*n* = 260) can be found in Table 2. The average age of the respondents in both neighbourhoods was 59 years old, and most of the respondents were female (respectively 62% and 71%). The family member questionnaire was filled out by 25 family members; demographic questions were only asked from family members of residents of one of the nursing homes. Therefore, we have not tabulated the responses.

**Table 1:** Characteristics of the staff members of the two nursing homes

	Nursing home 1(*n* = 18)	Nursing home 2 (*n* = 20)
Age	46.8 (sd = 14.1, range 21–63)	45.6 (sd = 13.5, range 20–65)
Sex, percentage female	94%	95%
Education		
- Intermediate vocational training	78%	80%
- Bachelor	6%	10%
- Other	17%	10%
Years working in this facility	10.6 (sd = 10.7, range:0–41; missing 1)	8.8 (sd = 10.7, range 0–30; missing 3)
Born in the village	28%	32% (1 missing)
Currently living in the village	44%	58% (1 missing)

**Table 2:** Characteristics of the inhabitants of the neighbourhoods surrounding the two nursing homes

	Nursing home 1 (*n* = 141)	Nursing home 2 (*n* = 119)
Age	59.9 (sd = 15.5, range 25–92; 2 missing)	59.8 (sd = 15.0, range 18–96)
Sex, percentage female	61.9%	71.1%
Education		
- Low	31.2%	16.0%
- Middle	37.7%	58.9%
- High	25.4%	22.3%
- Other	5.8%	2.7%
- Missing	3	7
Years living in this neighbourhood	33.2 (sd = 20.6, range 0–82)	25.2 (sd = 16.2, range 0–74, missing 17)

### Network connections between nursing staff and patients with dementia before admission to the nursing home

Ten out of 36 staff members (28%; 2 missing) said they knew residents with dementia already before their admission. These could be direct or indirect ties. Examples of direct contacts are:

- *“I knew Mr. C., he used to have a shop in the village; I also knew his sons”*
- *“I knew Mrs. W., she is my aunt. She has made many clothes for us on her sewing machine, as well as the first communion dress for my daughter”*

Indirect contacts exist when the staff member and the patient with dementia have a contact in common. Examples of indirect contacts are:

- *“I did not know Mr. Z. personally before admission, but his son in law had been my teacher”*
- *“ I did not know Mrs. W., but her daughter works here, she is a close colleague”*
- *“I did not know Mr. C. personally, but I knew his daughter”*

Some of the staff members also named multiple direct or indirect connections:

- *“Mrs. C. is my great aunt. In addition, I knew Mrs. Y., Mrs. O., Mrs. T., and Mrs. S. from residential care [before they developed dementia]”*
- *“One of the residents I knew personally and also the children of this resident. Some residents or family members knew my parents”*

**Fig. 1:**
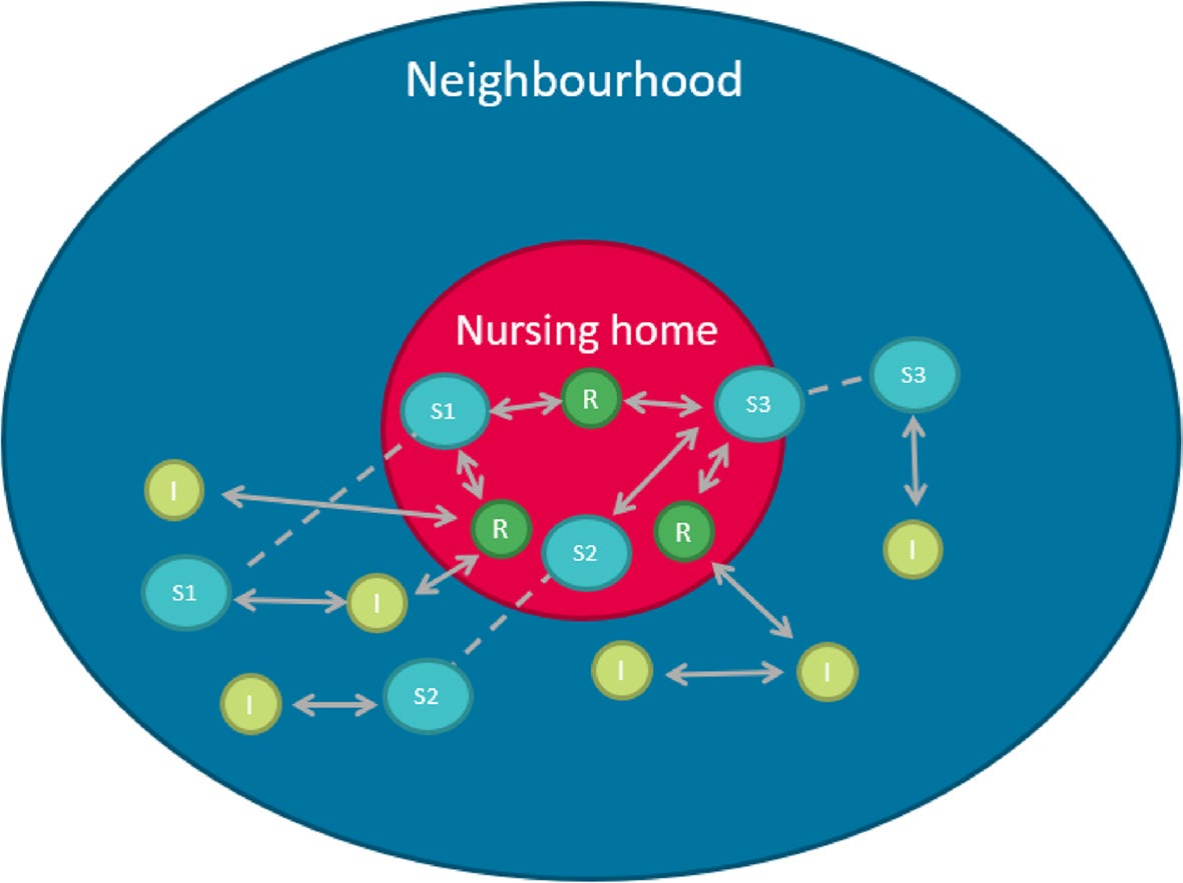
Pre-existing networks between residents and staff and indirect networks through family members and acquaintances of inhabitants of the neighbourhood. Note: dotted line indicates same person, i.e. staff members. Solid lines with arrows are relationships within or outside the nursing home (*R* = resident; S = staff; I = neighbourhood inhabitant)

We depict these networks in Fig. 1. The red circle represents the nursing home in which staff members (S) and residents (R) know each other, as depicted by the arrows. The blue circle represents the neighbourhood surrounding the nursing home, with the arrows describing connections between residents and neighbourhood inhabitants (I). Staff members also live in the neighbourhood. This is depicted by the dotted line: the dotted line between the S in the red and blue circle indicates that this is the same staff member, with the arrows indicating the connections between staff member and inhabitants in the community.

There are not only pre-existing links between nursing staff and residents with dementia, but also between family members of residents with dementia and nursing staff in the nursing homes. We have asked family members of residents about this. Nearly half of them (48%) have pre-existing relations with staff members. Some examples are:

- *“I [daughter of a resident with dementia of the nursing home] knew several staff members from the past, being former neighbours or just knowing them from the village”*
- *“One of the staff members lives in the same neighbourhood as me”*

We asked if nursing staff talk to family members and acquaintances of residents with dementia when they visited the ward. Almost all staff members (87%) reported that they frequently talked to family members and acquaintances on their visits to the facility. In addition to the health of and care provision to the residents with dementia, staff reported that they talk also about the family occasions in the life of family members, such as the birth of a grandchild. Almost 1 in 3 members of nursing staff (29%, 4 missing) also talked to family members outside work.

We asked nursing staff who lived in the village where the nursing home was located, if they are approached by neighbourhood inhabitants with positive or negative viewpoints about the facility. In total, 11 out of 37 (30%) of the staff members said they were approached sometimes or often about positive and negative aspects of the facility; for 26 staff members this happened seldom or never.

### Networks of nursing staff and the local community

Eleven staff members or nearly one third (30%; 1 missing) were born in the village where the nursing homes are located. Just over half (51%; 1 missing) is currently living in the village.

Many of the inhabitants of the neighbourhoods were born in or live already for quite some time in the neighbourhoods surrounding the two LTC facilities, on average 33 years in the neighbourhood around nursing home 1 and 25 years in the neighbourhood around nursing home 2 (Table 2).

Of the neighbourhood inhabitants, 22% (*n* = 56) know staff members of the dementia wards in the nursing homes; 36% (*n* = 20) of the persons responded that they talk to these staff members at least once a week. When we look at the total of personnel working in the nursing homes, the numbers are higher. Of the neighbourhood inhabitants, 53% (*n* = 134) know persons working in the nursing home. In two fifths of the cases (21%) the staff member is a neighbour; 13% is a family member; 24% a friend or acquaintance; 11% a (former) colleague; and in 33% there was another relation to the staff member. However, the possibility that people live in the same neighbourhood as staff members but do not consider them a direct neighbour should be considered.

**Fig. 2:**
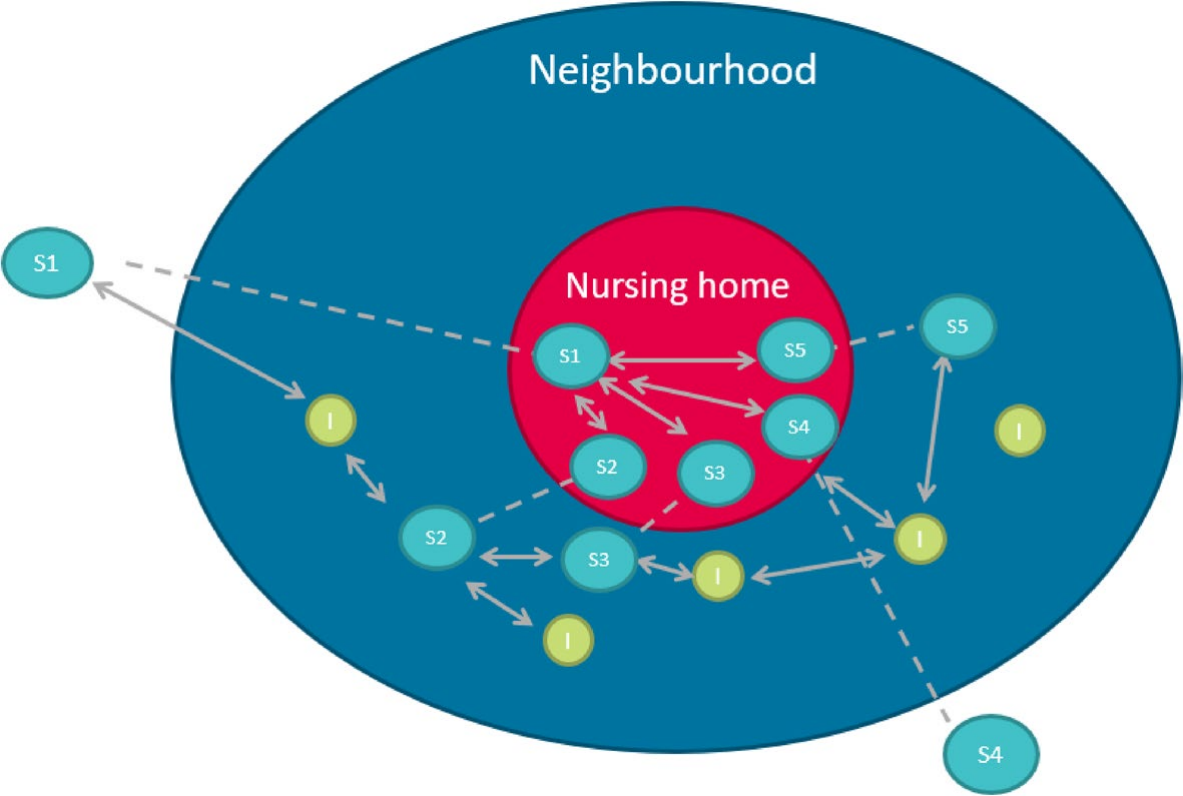
Neighbourhood embeddedness of nursing home staff. Note: dotted line indicates same staff member. Solid lines with arrows are relationships within or outside the nursing home (S = staff; I = neighbourhood inhabitant)

Eight percent of the neighbourhood inhabitants in our sample work as a volunteer in the nursing home. Figure 2 shows this aspect of embeddedness. The red circle represents the nursing home; the blue circle represents the neighbourhood surrounding the nursing home. Staff members who live in the neighbourhood and neighbourhood inhabitants may know each other, as depicted by the arrows. Not all staff members live in the neighbourhood, but can still have connections with neighbourhood inhabitants outside of work as is illustrated by staff member S1.

### Ties between neighbourhood inhabitants and residents of the nursing home

**Fig. 3:**
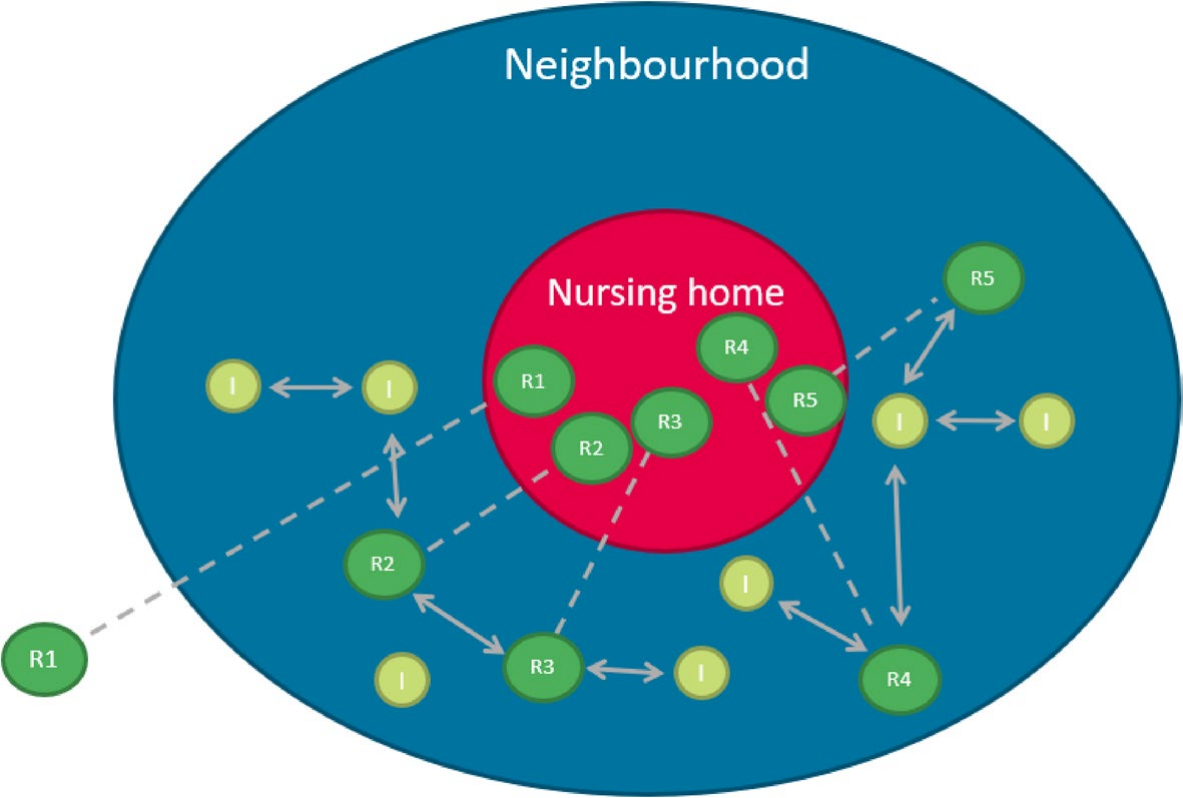
Neighbourhood network of residents. Residents used to live in the neighbourhood of the nursing home and may maintain existing ties. Note: dotted line indicates the same resident. Solid lines with arrows are relationships within or outside the nursing home (R = resident; I = neighbourhood inhabitant)

Two out of five inhabitants (42%) knew residents in the nursing home. Their relationship with residents of the nursing homes was in almost equal frequency a family member (30%) or friend (27%); in 20% of the cases, it concerns a neighbour; the rest are other relationships (including (former) colleagues). Given the fact that relatives and friends may also live in the neighbourhood, certainly more than 20% of the relations were linked to the common neighbourhood. Figure 3 schematises this aspect of embeddedness.

### Opportunities to meet residents and nursing staff via service provision

**Fig. 4:**
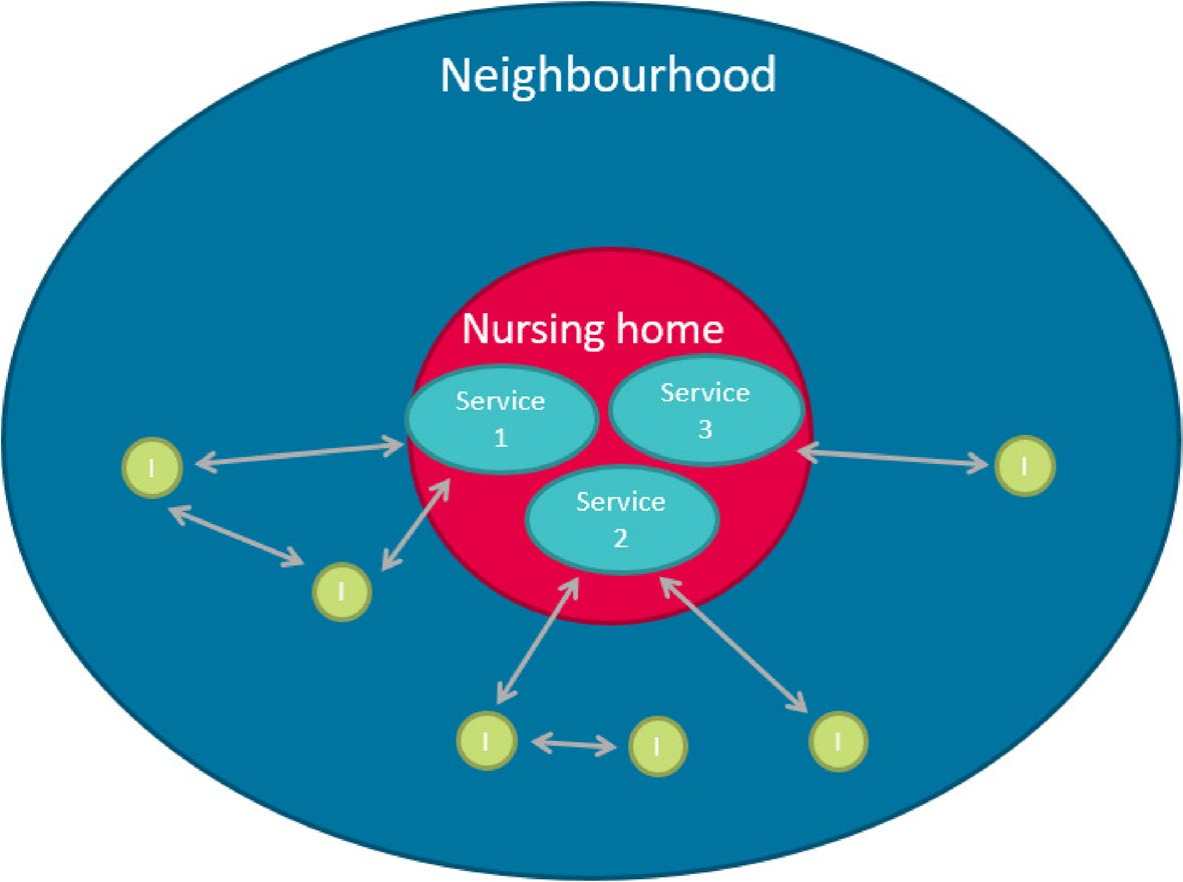
Service provision to neighbourhood inhabitants provides opportunities to meet residents and staff members. Note: Service 1,2,3 = service provided by the nursing home; I = neighbourhood inhabitant)

Close to half of the neighbourhood inhabitants (46%) visited the nursing home during the past 12 months. One third of those visiting the facility (32%) did so more than once per week. Over two thirds (38%) visited the nursing home for a visit to a relative or friend; a quarter (24%) for (voluntary) work. The two facilities differ in how they are located as well as in the services and amenities that are accessible for the people in the neighbourhood. One of the nursing homes lies adjacent to a park area that is used by local people for recreation, allotment gardens or to walk the dog, and there is a restaurant for residents and persons in the community. The location of the other nursing home lies in the centre of the village and, in addition to providing dementia care, also provides office space for other health care providers, such as a physiotherapist, and evening meals for residents living in the building and persons living nearby. Both locations also serve as polling station for local, regional and national elections. We asked neighbourhood inhabitants if they visited the nursing home apart from visiting residents or doing volunteer work. In nursing home 1, of the 66 respondents that visited the nursing home in the last 12 months, 34% used the park area surrounding the nursing home and 10% participated in nursing home activities. Also 10% of respondents ate in the restaurant. Incidental activities that were named were for instance the use of the polling station, and watching a game of billiards. In the other nursing home, of the 53 persons that visited the nursing home in the last 12 months, 26% visited a (physio)therapist, 9% visited their GP in the nursing home, 7% of respondents ate in the restaurant and 6% went to the hairdresser. Incidental activities that were named by respondents were again the use of the polling station an visiting he library. Figure 4 schematises this aspect of embeddedness.

## Discussion

As yet, little is known about social networks and embeddedness of LTC-facilities [22], such as nursing homes and our study give first insights in how nursing homes are embedded in the community. Studies in other healthcare organisations have shown the importance of studying social networks with regard to job outcomes such as job satisfaction [36] and job turnover [34].

Our description of the social embeddedness of nursing homes shows the existence of network connections between nursing staff on the one hand and the residents with dementia they care for and their relatives on the other hand. These connections have often been existing for long, and exceed the duration of time the residents live in the facility. Depending on the perspective, over a quarter (staff members) to nearly half (family members of residents) reported such pre-existing connections. Such connections that cross the boundaries of the nursing home are related to better quality of care [1]. At the background of these productive networks may be that staff members and neighbourhood inhabitants are members of local networks and are connected through direct and indirect social ties. The examples given by nursing staff give insight in the social roles or social identity of residents with dementia in the community. For instance, as seen in the results, by describing the former jobs of residents or activities that they carried out for the local community. The direct or indirect networks between staff and residents with dementia thus contain *information* that may help staff in caring for residents. This is particularly important for residents in dementia wards who are less able to communicate their wishes and worries. Interestingly, these descriptions may also help in *maintaining social citizenship* of residents with dementia, so that they are seen as individuals worthy to engage with by nursing staff, instead of marginalised members of society [4, 26], focusing not on the illness itself but on social inclusion instead [20].

Staff members and neighbourhood inhabitants also know each other and every now and then talk about the nursing home. Neighbourhood inhabitants visit residents with dementia and are perhaps able and motivated to *support* staff. The social networks of neighbourhood inhabitants and staff may in addition connect staff to the neighbourhood and through this connection also to their employment at the nursing home. The fact that these networks also contain residents, and that -as we found in our study- staff and inhabitants talk about work may provide staff with *recognition* for their work.

Our study contributes to the knowledge about the dynamic nature of neighbourhoods and the use of services. We found that neighbourhood inhabitants use different services in the nursing homes. It was not possible to determine if the use of these services were an effect of or a stimulant to social networks. Tulin et al. (2021) [27] discuss that more networks may not lead to more use of neighbourhood resources, but that the relationship may be the other way around: neighbourhood resources facilitate network connections in the neighbourhood. If so, this means that by offering service provisions to the neighbourhood, nursing homes contribute actively to network connections and cohesiveness in the neighbourhood. It is important to study this role of nursing homes further. Especially, as social networks and neighborhood attachment are directly related to feelings of loneliness in older adults [16]. Thus by increasing social embeddedness, nursing homes may contribute to healthy aging of all older persons in the local community and not only nursing home residents.

Service provision may also add to more familiarity with dementia care. By organizing dementia care in long-term care institutions, such as nursing homes and homes for the elderly over the past decades in the Netherlands, people with dementia have largely disappeared from the community. People in the community often don’t know how to interact with people with dementia and how to help them. They have become ill at ease in (accidental) meetings with people with dementia. Part of feeling at ease is what Blokland and Nast [3] have called public familiarity. The fact that people from the neighbouring residential areas see people with dementia, possibly greet them and see them interact with other people, makes people with dementia familiar again, with may lead to more *positive attitudes towards dementia*, which is in turn is a critical factor for ‘dementia friendly societies’ (see [13, 15, 21]).

Knowledge about the local networks around nursing homes, and more generally LTC facilities, is also important for other reasons. Unfortunately COVID- 19 has shown that connections between nursing staff and residents were also the major source for infections in nursing homes. In their study on social networks using data from smart phones Chen et al. [6] showed how nursing staff working in multiple nursing homes were related to the transmission of COVID- 19 between homes.

Local networks are also related to the recruitment and retainment of staff, one of the major challenges in many European countries, where different approaches are necessary for nursing homes due to different local situations [7].

Numerous contacts and relations of staff in the community are important for *job-embeddedness* [24]. Staff tenure is high in our study, at least measured from how many years they already work in the nursing home. However, this may be both a cause and a consequence of embeddedness. In particular in wards with people with dementia, personal continuity in the persons of the staff members may be important. There is also a potential for help, resulting in neighbourhood inhabitants acting as volunteers in the nursing homes. The review of Handley et al. [10] states that ‘a considerable investment is needed to initiate and maintain volunteering initiatives, but there are positive benefits for volunteers, residents and staff if an intervention is sustained’. The existing social connections and perceived support may thus be possible resources to increase the number of volunteers and positive outcomes for residents and staff.

Based on these findings, we formulate different hypotheses on how performance of nursing homes may be improved. First, on the level of care provision, our hypothesis is that quality of care is enhanced by social networks through information exchange about residents and support for staff members.

Secondly, on the level of the community, we hypothesize that social networks aid in maintaining social citizenship of residents and positive attitudes towards dementia among local inhabitants. As we have seen that nursing homes are part of the community through numerous ties, we expect that both aspects will influence social inclusion and thus performance of nursing homes and LTC facilities in general.

Finally, we hypothesize that that social networks positively influence job embeddedness, leading to more tenure of staff and more participation of volunteers.

Future research needs to elaborate and test these hypotheses, preferably in a longitudinal and/or comparative design.

Our description of the social embeddedness of nursing homes is particularly relevant in view of recent policy changes in the Netherlands, especially the devolution of care and support to municipalities. These policies place more emphasis on living longer independently, with support from family, neighbours, and other persons in the social networks of the older person. Consequently, people with dementia will be living in the community more often in the near future. Many people with dementia, however, will still live in a nursing home at some stage of their life. It is a societal challenge to provide long-term care for people with dementia in a way that fosters their rights to have a high quality of life, including as much autonomy as possible in an environment that is safe and stimulating.

### Strengths and limitations of our study

To our best knowledge, our study is the first to describe the embeddedness of nursing homes in local social networks. We have approached embeddedness from different perspectives, asking questions to staff members, family members of nursing home residents with dementia, and inhabitants of the surrounding neighbourhoods.

Given the new and exploratory character of our study, we compared only two nursing homes and the surrounding networks in relatively small communities. The situation in urban areas is different in terms of the stability of the inhabitants of the neighbourhoods, the recruitment of personnel and the place where residents of the facilities used to live. However, also urban nursing homes have to connect to the surrounding neighbourhoods and are developing policies to do so. For instance, by making visiting the nursing home more attractive for neighbourhood inhabitants by co-locating other services and amenities.

In addition, studies should be carried out in other countries and geographical areas. The Netherlands is a relatively small and densely populated country with in total 342 municipalities. The majority of older persons go to a nursing home in in the same municipality. About 35% of residents move to a nursing home in a different municipality than their previous home [33]. Geographical distances when moving into a nursing home are therefore small. It is crucial to study social networks and organisational embeddedness in countries where geographical distances are different and family and friends may be located further away.

A weakness of our study is that we don’t have information on full networks and on the strength of network ties, but only on the ties, reported from different perspectives. We were therefore not able to analyse the structural aspects of the networks, such as density, which may also be important for the performance of organisations [25]. It was -due to the restrictions of this study- also not possible to consider the strength of ties. More studies on direct and indirect ties and how they relate to nursing home care are necessary.

We used paper surveys which we distributed in the neighbourhood of the nursing home as we did not have names of email addresses of neighbourhood inhabitant. Therefore, we do not know if an electronic survey may have received more response. Further research using other methods for collecting data in neighbourhoods would be useful. In addition, the response rates were lower for family members. This may be due to the fact that we sent the questionnaire specifically to the legal representative of the residents with dementia. This is often, but certainly not always the person who has the closest relationship with the resident. However, we deemed this approach most suitable as the legal representative was also asked to give informed consent for this study.

Yet, although our numbers of participant were relatively small, our results show the usefulness of studying networks from different perspectives at one moment in time using questionnaires. Further studies can use this approach.

## Conclusions

In conclusion, nursing homes are linked to their social environment through social networks. Neighbourhood inhabitants are also family members of residents with dementia and several of the inhabitants work as staff members in the facility and know residents and their family members through community connections. These connections form extensive networks that cross the boundary of the nursing home and may exist over a long period of time. These networks constitute the social resources of the facility and can be employed to improve quality of care for older people, in particular those with dementia. Some of these relationships can be supported by active policies of the nursing homes and the local authorities. Expanding nursing homes to new locations or the decision to re-locate facilities may take into account the network resources that will be destroyed or can be activated at a given or future location.

## Supplementary Information

Supplementary Material 1.

## Data Availability

The datasets analysed during the current study are not publicly available due to privacy reasons, but are available from the corresponding author on reasonable request.

## Abbreviation

LTC: Long-term care
