# Supplementary material for: Social networks of neighbourhood inhabitants, residents of a care facility, and nursing staff: a case study in two long-term care facilities in the Netherlands
Source: BMC Geriatr. 2025 May 8;25:316. doi: 10.1186/s12877-025-05948-z (PMC12060523; doi:10.1186/s12877-025-05948-z)
Supplement: Supplementary file 1 — Supplementary Material 1. [file 12877_2025_5948_MOESM1_ESM.docx]

**Appendix 11: Questionnaire for nursing staff**

| General data |
| --- |
| What is your age? |
| What is your gender? |
| What is your highest received education?   - Low - Middle - High - Other |
| What is your job function?   - Care worker - Welfare worker - Living assistent |
| Connections with residents with dementia |
| We would like to know whether you already knew residents before their admission to this department. Or perhaps you already knew the residents’ family, friends or acquittances before the residents was admitted to this department.  Think of the residents in this department. How many residents did you know personally or through others in your own circle? If you knew residents directly or through, for example, family, friends or acquittances, would you please not this below as in the following examples?  Some examples:   - Example 1: I did not know Resident Y personally before the admission. However, I went to high school with her daughter. - Example 2: My father knows Residents X from the past, they used to play billiards club together. He sometimes came to visit my parents.   You can describe the relation to the residents below (as in the examples). If you did not know any residents before admission, you can tick the box below.   - I did not know any residents before their admission to this department. |
| Contact with family members and acquittances of residents |
| Do you talk to family members or acquittances of residents when they visit the ward?   - Never - Barely - Sometimes - Regularly - Often |
| What do you talk about? (Multiple answers possible)   - Health of the resident - Well-being of the resident - Communication with the family of the resident - (Leisure) activities - Habits and hobbies of the resident - Safety on the ward - Cooperation on the ward - Other |
| Do you speak with family members or acquittances of residents about the care provided in [name nursing home] on other occasions outside of work?   - Never - Barely - Sometimes - Regularly - Often |
| Contact with community members of [name nursing home] |
| Are you born in [name municipality]?   - Yes - No |
| Do you currently live in [name municipality]?   - Yes - No |
| Are you ever approached by local residents about positive things they hear about [name nursing home]?   - Never - Barely - Sometimes - Regularly - Often - Always |
| Are you ever approached by local residents about negative things they hear about [name nursing home]?   - Never - Barely - Sometimes - Regularly - Often - Always |

**Appendix 2: Questionnaire for neighbourhood inhabitants**

| Questions about [name nursing home] |
| --- |
| Have you visited [name nursing home] in the past 12 months?   - Yes - no |
| How often do you visit [name nursing home]   - Every day - One or multiple times per week - One or multiples times per month - Less that once per month |
| What is your main purpose when you visit [name nursing home] (multiple answers possible)   - Visiting family/friends - Work/volunteer work - Recreative use of the outdoor area (walking, communal garden maintenance) - Using the outdoor area to pass through - Participating in activities - Eating at the restaurant - Other, namely.. |
| Do you know persons that live in [name nursing home]   - Yes - No |
| What is your relationship with these person(s)? (If you know multiple persons living in [name nursing home], you can choose multiple answers)   - Partner - Father/mother - Brother/sister - Uncle/aunt - Cousin - Other relative - Friend or acquittance - Neighbour of community member - (Former) colleague - Other connection |
| How often do you visit these persons? (If you know multiple persons living in [name nursing home], you can choose multiple answers)   - Every day - One or multiple times per week - One or multiples times per month - Less that once per month - Never |
| Do you know persons that work at [name nursing home]?   - Yes - No |
| What is your relationship with these person(s)? (If you know multiple persons living in [name nursing home], you can choose multiple answers)   - Family member - Friend or acquittance - Neighbour or community member - (Former) colleague - Other connection |
| Are you an active volunteer at [name nursing home]?   - Yes, namely… - No |
| Experiences with dementia |
| Do you know a person with dementia?   - Yes - No |
| What is your relationship with these person(s)? (If you know multiple persons with dementia, you can choose multiple answers)   - Partner - Father/mother - Brother/sister - Uncle/aunt - Cousin - Other relative - Friend or acquittance - Neighbour of community member - (Former) colleague - Other connection |
| How often do you contact this person? (If you know multiple persons with dementia, fill in this question for the person nearest to you)   - Every day - One or multiple times per week - One or multiples times per month - Less that once per month - Never |
| Do you know persons working in dementia care?   - Yes - No |
| Do you know persons working in dementia care [name nursing home]?   - Yes - No |
| How often do you contact these persons? (If you know multiple persons working in [name nursing home], you can choose multiple answers)   - Every day - One or multiple times per week - One or multiples times per month - Less that once per month - Never |
| Do you talk with these persons about [name nursing home]   - Yes - No |
| Would you ever approach these person(s) about positive things you hear about [name nursing home]?   - Never - Barely - Sometimes - Regularly - Often - Always |
| Would you ever approach these person(s) about negative things you hear about [name nursing home]?   - Never - Barely - Sometimes - Regularly - Often - Always |
| Background questions |
| Were you born in [name municipality]?   - Yes - No |
| How long have you lived in this neighbourhood?  … Years |
| What is your gender? |
| What is your age? |
| What is your highest completed education? |

**Appendix 3: Questionnaire for family members**

| Questions about your relative (with dementia) |
| --- |
| Date of birth |
| Gender |
| Zip code |
| Highest completed education |
| Marital status |
| Children |
| Questions for family member |
| How often do you speak with care professionals when you visit your relative at [name nursing home]?   - Hardly - Barely - Sometimes - Regularly - Often |
| If so, what do you talk about? (Multiple answers possible)   - Health of the resident - Well-being of the resident - Communication with the family of the resident - (Leisure) activities - Habits and hobbies of the resident - Safety on the ward - Cooperation on the ward - Other |
| Do you speak with care professionals of your relative on other occasions outside of [name nursing home]?   - Never - Barely - Sometimes - Regularly - Often |
| Contact with care professionals of [name nursing home] |
| We would like to know whether you already knew care professionals before the admission of your relative to [name nursing home]. Or perhaps you already knew the care professionals’ family, friends or acquittances before your relative was admitted to [name nursing home].  Think of the care professionals on the department where your relative lives. How many care professionals did you know personally or through others in your own circle? If you knew care professionals directly or through, for example, family, friends or acquittances, would you please not this below as in the following examples?  Some examples:   - Example 1: I did not know Care professional Y personally before the admission. However, I did know her aunt. - Example 2: My sister knows Care professional X from the past, they used to do gymnastics together. She sometimes came to visit us in our home.   You can describe the relation to the residents below (as in the examples). If you did not know any residents before admission, you can tick the box below.   - I did not know any care professional before their admission to this department. |
| Background questions |
| Were you born in [name municipality]?   - Yes - No |
| Do your currently live in [name municipality]? |
| - Yes - No |
